# Effects of lenalidomide on the bone marrow microenvironment in acute myeloid leukemia: Translational analysis of the HOVON103 AML/SAKK30/10 Swiss trial cohort

**DOI:** 10.1007/s00277-021-04467-2

**Published:** 2021-03-02

**Authors:** Magdalena M. Brune, Georg Stüssi, Pontus Lundberg, Visar Vela, Dominik Heim, Markus G. Manz, Eugenia Haralambieva, Thomas Pabst, Yara Banz, Mario Bargetzi, Rainer Grobholz, Martin Fehr, Sergio Cogliatti, Gert J. Ossenkoppele, Bob Löwenberg, Christina Biaggi Rudolf, Qiyu Li, Jakob Passweg, Luca Mazzuchelli, Michael Medinger, Alexandar Tzankov, on behalf of the Dutch-Belgian Hemato-Oncology Cooperative Group (HOVON) and Swiss Group for Clinical Cancer Research (SAKK)

**Affiliations:** 1 Institute of Medical Genetics and Pathology, University Hospital Basel, Schoenbeinstrasse 40, 4031 Basel, Switzerland; 2 Oncological Institute of Italian Switzerland, Via Ospedale, 6500 Bellinzona, Switzerland; 3 Division of Hematology, Department of Medicine, University Hospital Basel, Petersgraben 4, 4031 Basel, Switzerland; 4 Department of Medical Oncology and Hematology, University and University Hospital Zurich, Raemistrasse 100, 8091 Zurich, Switzerland; 5 Institute of Pathology and Molecular Pathology, University Hospital Zurich, Schmelzbergstrasse 12, 8091 Zürich, Switzerland; 6 Department of Medical Oncology, Inselspital, University Hospital and University of Bern, 3010 Bern, Switzerland; 7 Institute of Pathology, University of Bern, Murtenstrasse 31, 3008 Bern, Switzerland; 8 Clinic for Oncology, Hematology and Transfusion Medicine, Cantonal Hospital Aarau AG, Tellstrasse 25, 5001 Aarau, Switzerland; 9 Institute of Pathology, Cantonal Hospital Aarau AG, Tellstrasse 25, 5001 Aarau, Switzerland; 10 Clinic for Medical Oncology and Hematology, Cantonal Hospital St. Gallen, Rorschacher Strasse 95, 9007 St. Gallen, Switzerland; 11 Institute of Pathology, Cantonal Hospital St. Gallen, Rorschacher Strasse 95, 9007 St. Gallen, Switzerland; 12 Department of Hematology, Amsterdam University Medical Center, Cancer Center Amsterdam, VU University Medical Center, De Boelelaan 1118, 1182 Amsterdam, DB Netherlands; 13 Department of Hematology, Erasmus University Rotterdam, Dr. Molewaterplein 50, 3000 Rotterdam, CA Netherlands; 14 Swiss Group for Clinical Cancer Research (SAKK), Coordinating Center, Effingerstrasse 33, CH-3008 Bern, Switzerland; 15 Cantonal Institute of Pathology, Via Selva 24, 6601 Locarno, Switzerland; 16 Division of Internal Medicine, Department of Medicine, University Hospital Basel, Petersgraben 4, 4031 Basel, Switzerland

**Keywords:** Acute myeloid leukemia, Bone marrow microenvironment, Cereblon, Lenalidomide, Microvessel density, T cells

## Abstract

This translational study aimed at gaining insight into the effects of lenalidomide in acute myeloid leukemia (AML). Forty-one AML patients aged 66 or older of the Swiss cohort of the HOVON-103 AML/SAKK30/10 study were included. After randomization, they received standard induction chemotherapy with or without lenalidomide. Bone marrow biopsies at diagnosis and before the 2nd induction cycle were obtained to assess the therapeutic impact on leukemic blasts and microenvironment. Increased bone marrow angiogenesis, as assessed by microvessel density (MVD), was found at AML diagnosis and differed significantly between the WHO categories. Morphological analysis revealed a higher initial MVD in AML with myelodysplasia-related changes (AML-MRC) and a more substantial decrease of microvascularization after lenalidomide exposure. A slight increase of T-bet-positive TH1-equivalents was identifiable under lenalidomide. In the subgroup of patients with AML-MRC, the progression-free survival differed between the two treatment regimens, showing a potential but not significant benefit of lenalidomide. We found no correlation between the cereblon genotype (the target of lenalidomide) and treatment response or prognosis. In conclusion, addition of lenalidomide may be beneficial to elderly patients suffering from AML-MRC, where it leads to a reduction of microvascularization and, probably, to an intensified specific T cell-driven anti-leukemic response.

**Supplementary Information:**

The online version contains supplementary material available at 10.1007/s00277-021-04467-2.

## Introduction

Most individuals with acute myeloid leukemia (AML) are older than 65 years upon diagnosis [1]. As the incidence of unfavorable genetic alterations increases with age, the prognosis of AML in the elderly is dismal and associated with the worst median overall survival (OS) of all cancers in this age group with nearly 80% of the patients died after 1 year [1, 2]. Aggravatingly, chemotherapeutic treatment remains challenging due to the rising incidence of comorbidities and the poorer performance status of aged individuals. Although the development of less toxic and more effective treatment options is of utter interest, only modest progress has been achieved in clinical outcomes of elderly AML patients in the last decade.

Based on its clinical activity in related disorders such as myelodysplastic syndromes (MDS) and other hematologic malignancies including multiple myeloma and follicular lymphoma [3–5], the orally active immune-modulatory drug (IMiD) lenalidomide gathered attention as a novel anti-neoplastic agent for the treatment of AML. Lenalidomide targets the omnipresent E3 ubiquitin ligase complex cereblon [6], which mediates its effects on tumor cells and non-neoplastic cells of the tumor microenvironment [7]. Lenalidomide activates cereblon’s ligase activity leading to faster degradation of the transcription factors Ikaros and Aiolos, which play an important role in the regulation of B- and T cell development [7], and the casein kinase 1A1 (CK1alpha), which is a negative regulator of p53 [8]. CK1alpha is encoded by the *CSNK1A1* gene, which can be deleted or mutated in del(5q) MDS. In murine models, haploinsufficiency of this gene leads to hematopoietic stem cell expansion, whereas a complete loss induces stem cell apoptosis by activation of p53, explaining at least partially the effect of lenalidomide in del(5q) MDS [9]. In analogy, *Csnk1a1* knockdown in AML cell lines increases p53 activity and myeloid differentiation and results in selective elimination of leukemic cells [10].

Furthermore, autoubiquitination (and thus degradation) of wild-type cereblon is prevented by lenalidomide. In net terms, lenalidomide has anti-proliferative effects particularly on malignant B-cells and stimulating effects on by-stander T cells and natural killer (NK) cells, while promoting the production of anti-inflammatory cytokines [7]. Next to this anti-neoplastic and immune-modulatory effect, lenalidomide impairs the secretion of the vascular endothelial growth factor (VEGF) in the bone marrow stroma, eventually influencing vessel density and other microenvironmental changes [11]. Recently, the HOVON/SAKK study group published their data of the HOVON103 AML/SAKK 30/10 trial on the addition of lenalidomide to standard intensive treatment in elderly patients with AML and high-risk MDS [12]. Unfortunately, the study failed to show a clear benefit for those patients receiving additional lenalidomide, putting it in line with many other surveys performed in the setting of AML in the elderly. Here, we present a translational research analysis of the study encompassing patients of the Swiss study cohort, for whom bone marrow biopsies at study inclusion and—for the majority of individuals—before the 2nd induction cycle were available. Our results suggest that addition of lenalidomide to induction chemotherapy may be beneficial to elderly patients suffering from AML with myelodysplasia-related changes (AML-MRC).

## Materials and methods

### Patient cohort and treatment

Forty-one patients of the Swiss cohort of HOVON103 AML/SAKK 30/10 trial were included in this translational research study, of whom 20 were male and 21 female (Table 1). They were all previously untreated, aged ≥ 66, had a WHO performance score of ≤ 2, and a morphologically confirmed diagnosis of de novo AML. Patients with acute promyelocytic leukemia were not included. The patients’ mean age at first diagnosis was 69 years (range 66 to 76). Clinical outcome parameters for this study were progression-free survival (PFS) and overall survival (OS). For further details, we refer to the HOVON/SAKK study group publication on the clinical trial [12]. This study was approved by the ethics committee of Northwestern Switzerland (EKNZ BASEC 2016-01218).

**Table 1:** Patients’ characteristics and responses to treatment

	Standard treatment (*n* = 19): *n*(%)	With lenalidomide (*n* = 22): *n*(%)
Sex		
• Male	10 (52.6%)	10 (45.5%)
• Female	9 (47.4%)	12 (54.5%)
Dose of lenalidomide		
• 15 mg	NA	1 (4.5%)
• 20 mg	NA	21 (95.5%)
WHO classification of AML		
• AML NOS	10 (52.6%)	11 (50.0%)
• AML mutations	6 (31.6%)	4 (18.2%)
• AML specific translocations	2 (10.5%)	0 (0.0%)
• AML MRC	1 (5.3%)	7 (31.8%)
Age at registration (years)		
• 66–70	13 (68.4%)	20 (90.9%)
• 71–76	6 (31.6%)	2 (9.1%)
Karyotype according to Grimwade		
• Favorable	2 (10.5%)	0 (0.0%)
• Intermediate	15 (78.9%)	15 (68.2%)
• Adverse	0 (0.0%)	5 (22.7%)
• Missing	2 (10.5%)	2 (9.1%)
Best response after cycle 1		
• CR	11 (57.9%)	11 (50.0%)
• CRi	0 (0.0%)	4 (18.2%)
• PR	3 (15.8%)	0 (0.0%)
• RD	3 (15.8%)	6 (27.3%)
• Death in aplasia	0 (0.0%)	1 (4.5%)
• Death of indeterminate cause	2 (10.5%)	0 (0.0%)
Did patient start cycle 2?		
• No	4 (21.1%)	8 (36.4%)
• Yes	15 (78.9%)	14 (63.6%)
Best response after cycle 2 (only for patients started cycle 2)		
• CR	12	8
• CRi	1	3
• PR	1	1
• RD	1	0
• death in aplasia	0	1
• death of indeterminate cause	0	1

*NA*, not applicable; *NOS*, not otherwise specified; *MRC*, myelodysplasia-related changes; *CR*, complete remission; *CRi*, CR with incomplete hematologic recovery; *PR*, partial remission; *RD*, refractory disease

To morphologically assess the therapeutic impact on blasts and microenvironment, bone marrow biopsies were gained at the time-point of diagnosis and, whenever possible, before the beginning of the 2nd induction cycle.

Karyotypes were classified according to Grimwade et al. [13] into three prognostically relevant groups (favorable, intermediate, adverse). Considering morphological and genetical criteria, patients were subgrouped into different diagnostic categories, according to the current WHO classification of tumors of hematopoietic and lymphoid tissues [14].

As described in more detail by Ossenkoppele et al. [12], the patients randomly received either a standard remission induction regimen with or without lenalidomide. Of our cohort, 19 patients were assigned to the standard treatment arm (daunorubicin 45 mg/m^2^ days 1–3 and cytarabine 200 mg/m^2^ days 1–7 in cycle I; and cytarabine 1000 mg/m^2^ q 12 h days 1–6 in cycle II), and 22 patients additionally received lenalidomide at an assigned dose level (10 to 20 mg/day orally, days 1–21 of each cycle) (Table 1).

### Morphological, immunohistochemical, and molecular work-up of bone marrow biopsies

Bone marrow biopsies at the time-point of diagnosis were available in 39/41 cases. A second bone marrow biopsy, which was obtained before the 2nd induction cycle, was available in 28/41 cases. The specimens were fixed in 4% formalin and paraffin-embedded, followed by decalcification with ethylenediaminetetraacetic acid (EDTA) [15]. Hematoxylin-and-eosin- (H&E) and Gömöri-stained slides were reviewed, and the presence and amount of leukemic blasts, myelodysplasia-related changes, and the degree of myelofibrosis [16] were assessed. Immunohistochemistry was performed using the automated staining system Benchmark XT (Roche/Ventana Medical Systems, Tucson, USA). To evaluate microvessels, CD34 staining was performed and scored as described [17], and in cases with excessive amounts of CD34 positive blasts hampering quantification, supplemented by a CD31 staining. Stem cell niches were quantified using a nestin staining as previously shown [18]. Blast quantification was based on morphological analysis of the H&E slides corroborated by CD34 staining, if expressed by the tumor cells, and correlated with the blast counts assessed on aspiration smears. Erythropoiesis was investigated with the help of E-cadherin. Characterization and quantification of B-, T-, NK- cells, and monocytes were performed utilizing antibodies against CD3, CD4, CD8, CD20, CD56, CD57, FoxP3, granzyme B, PD1, PD-L1, TIA1, and T-bet as described [19, 20]. Lenalidomide’s target cereblon has also been stained for and was assessed depending on its intensity: a quality score from 0 (negative) to 3 (strong, unequivocal positivity) has been assigned. To highlight vascular endothelial growth factor (VEGF) and -receptor (VEGFR) expression alterations, stainings for VEGF and VEGFR2 were performed [17]. Antibody sources, dilutions, incubation, and retrieval conditions as well as cutoff scores are displayed in Table 2. Two authors (MMB and AT) investigated all stained slides, and reproducibility was estimated applying the Cronbach’s Alpha method.

**Table 2:** Antibodies applied and cut-off scores

Antibody	Source and clone or ID	Dilution	Scoring/counting
Cereblon	Celgene Corporation	1:400	Moderate to strong expression in > 50% of tumor cells
CD3	Ventana 790-4341	Ready to use	Any lymphocyte, finally scored as % positive cells/all cells
CD4	Cell Marque SP35	1:100	Any lymphocyte, finally scored as % positive cells/all cells
CD8	DAKO C8/144B	1:400	Any lymphocyte, finally scored as % positive cells/all cells
CD20	Ventana QBEnd/10	Ready to use	Any lymphocyte, finally scored as % positive cells/all cells
CD31	Ventana 760-4378	rReady to use	Any lymphocyte, finally scored as % positive cells/all cells
CD34	Ventana 790-2927	Ready to use	Any microvessel and any blast, finally scored as N^micorvessels^/mm^2^ or % positive blasts/all cells
CD56	Ventana 790-4465	Ready to use	Any lymphocyte, finally scored as % positive cells/all cells
CD57	Ventana 760-2626.	Ready to use	Any lymphocyte, finally scored as % positive cells/all cells
E-cadherin	Ventana EP700Y	Ready to use	Any erythropoietic cells, finally scored as % positive cells/all cells
FoxP3	Abcam mAbcam 22510	1:50	Any lymphocyte, finally scored as % positive cells/all cells
Granzyme B	Novocastra 11F1	1:100	Any lymphocyte, finally scored as % positive cells/all cells
Nestin	AbD Serotec 10C2	1:200	Any perivascular niche (either single cells or clusters of up to three cells), finally scored as N^niches^/mm^2^
PD1	Cell Marque NAT105	1:50	Any lymphocyte, finally scored as % positive cells/all cells
PDL1	Cell signaling E1L3N	1:50	Single+ cells, 1–5% + cells, or > 5% + mononuclear cells
T-bet	Abcam ab154200	1:100	Any lymphocyte, finally scored as % positive cells/all cells
TIA1	Biocare CM130C	1:25	Any lymphocyte, finally scored as % positive cells/all cells
VEGF	DAKO VG1	1:40 *	Moderate to strong expression in > 50% of tumor cells
VEGFR2	Neomarkers RB-10453-P1	1:10 *	Moderate to strong expression in > 50% of tumor cells

In all instances except for *, in which high pH buffers have been applied, respectively, antigen retrieval was based on lower pH buffers and microwaving

### Cereblon genotyping

DNA was extracted from 34 available bone marrow biopsies at the time of first diagnosis using the GeneReadTM DNA-FFPE-Kit (Qiagen, Hilden, Germany) according to the manufacturer’s protocol. Fifty nanograms of the extracted DNA was used to determine the rs1672753 (A/G) polymorphism located in the 5′ UTR of *CRBN*. Genotyping was performed using the Biorad QX200 digital PCR platform (Bio-Rad, Berkeley, CA, USA).

### Statistical analysis

All statistical analyses were performed with the IBM SPSS 25.0 (IBM, Armonk, New York) and R version 3.5.3. The degree of inter-observer consensus was evaluated by interclass correlation coefficients, using reliability Cronbach’s Alpha analysis, *α* values > 0.75 indicating a good agreement [21]. Comparisons were performed using the Kruskal–Wallis- or the Mann–Whitney *U* (MWU)–tests. Wilcoxon signed rank test was used to compare numeric variables between pre- and post-treatment. To investigate the correlation between two markers, Spearman rank correlation coefficient (*ρ*) was estimated; only the estimated *ρ* > 0.40 were further considered. The 95% confidence interval (CI) of *ρ* was based on bootstrap method. Progression-free survival (PFS) was defined as the time from registration until relapse or death, whichever occurred earlier. Overall survival (OS) was defined as time from registration until death. These time-to-event endpoints were analyzed using 50 ng Kaplan-Meier estimate, and 95% CI of its median was based on log transformation. Generally, log rank test was used to compare time-to-event endpoints between groups. However, if a small group (group size ≤ 2) was involved, permutation test was used. Cox proportional hazards regression model was used to investigate the association between time-to-event endpoints and continuous variables. If the distribution of continuous variable is not symmetric, it will be log transformed before modelling. *P* values < 0.05 were considered as significant. Two-sided tests were used throughout. All results were not corrected for multiple testing.

## Results

### Patient cohort and treatment

Patients’ baseline characteristics are shown in Table 1. Regarding the karyotype analysis according to Grimwade, two patients were categorized as having a favorable karyotype, five patients had a karyotype with adverse prognostic impact and the karyotype of 30 patients was classified as intermediate; in 4 patients, this information was missing. Patients were assigned to the treatment arms irrespective of their karyotype. Due to the random distribution, all patients with a favorable karyotype were allocated in the standard treatment arm, whereas all patients with an unfavorable karyotype received additional lenalidomide. The 30 patients with intermediate karyotype were distributed equally between both treatment groups.

According to the WHO classification 2017, 21 patients were categorized as AML, not otherwise specified (NOS) (AML, NOS). Of these, ten received standard treatment and eleven additional lenalidomide. AML with defining mutations (*NPM1*, *FLT3*, or *CEBPA)* applied to 10 patients, of whom 4 received lenalidomide and 6 did not. Specific translocations or inversions were found in another 2 patients [inv(16) or t(16;16)], who were assigned to the category AML with defining translocations; both patients underwent standard treatment. The AML category with myelodysplasia-related changes (AML-MRC), either histomorphologically or genetically, applied to 8 patients; 7 of them were treated with additional lenalidomide and only one with the standard regimen (Table 1).

### Morphological, immunohistochemical, and molecular work-up of pre- and post-treatment bone marrow biopsies

Internal consistency analysis regarding evaluation of the immunohistochemical markers yielded good or excellent results for myelofibrosis, nestin niches, CD34-positive blast counts, and counting of granzyme B, T-bet, FoxP3, CD3, CD4, CD8, and E-cadherin positive cells. Acceptable results were obtained for the analysis of microvessel density, PD-L1, CD20, and CD57. The reproducibility of PD1 was estimated as questionable and was poor for TIA1, and therefore no further analyses linked to this latter marker were done. CD56 staining never yielded positive cells, except for osteoblasts.

**Fig. 1:**
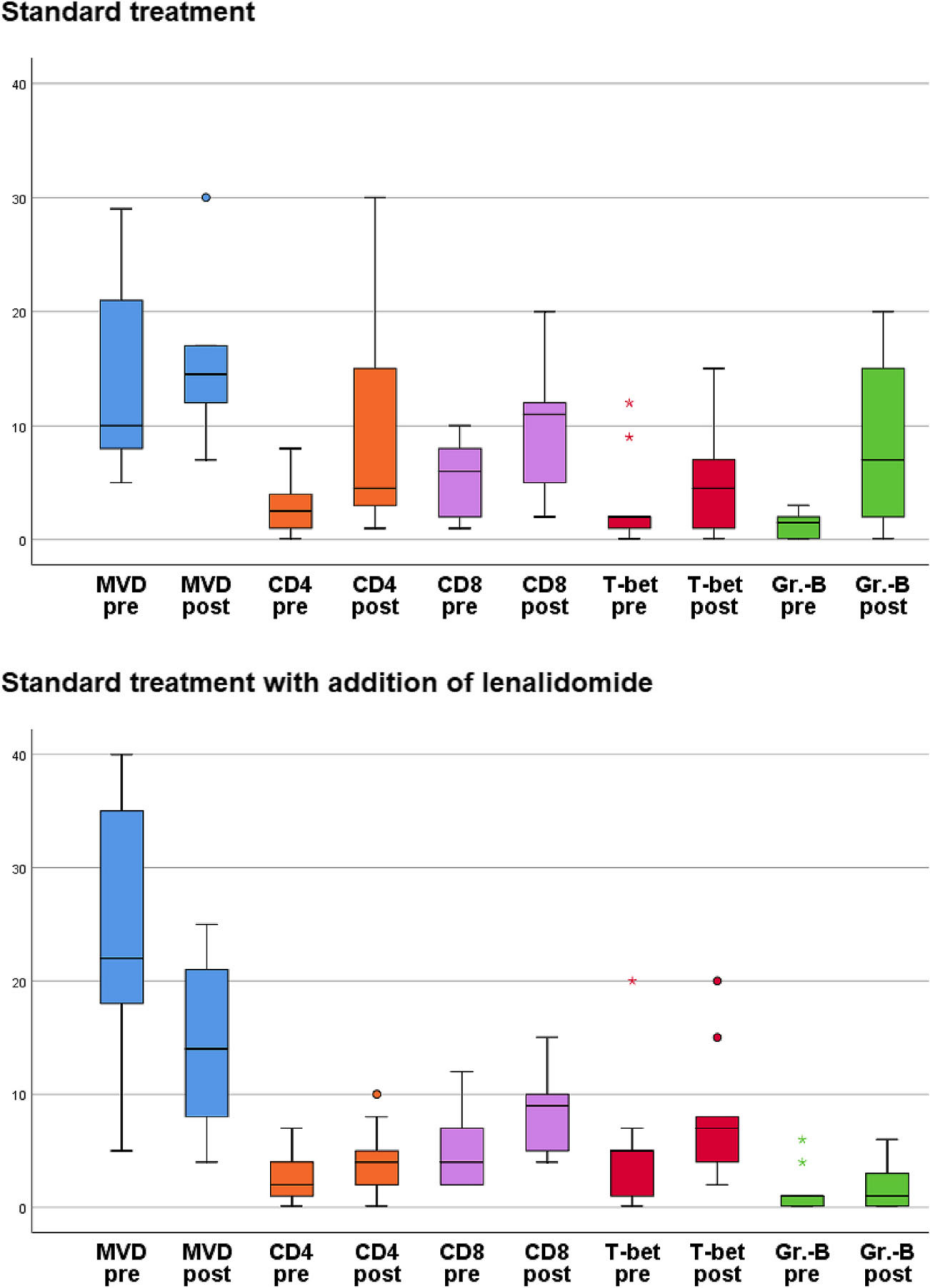
Boxplot diagram visualizing quantitative changes of microvessel density (MVD) and selected studied T cell populations in acute myeloid leukemia treated by either standard chemotherapy (upper) or standard chemotherapy and additional lenalidomide (lower); boxes are color coded according to variables and pairwise grouped before (pre) and after (post) treatment. Note the considerably more pronounced decrease of MVD and the more limited increase of T cells under lenalidomide with the exception of the T-bet-positive subpopulation that seems to more stringently increase with addition of lenalidomide

All applied immunohistochemical markers were analyzed regarding their distribution among the individual WHO categories, their prognostic impact, and under consideration of the administered therapy (Fig. 1 and Supplementary Table 1). Here, only potentially relevant results are described.

### Blasts

**Fig. 2.**
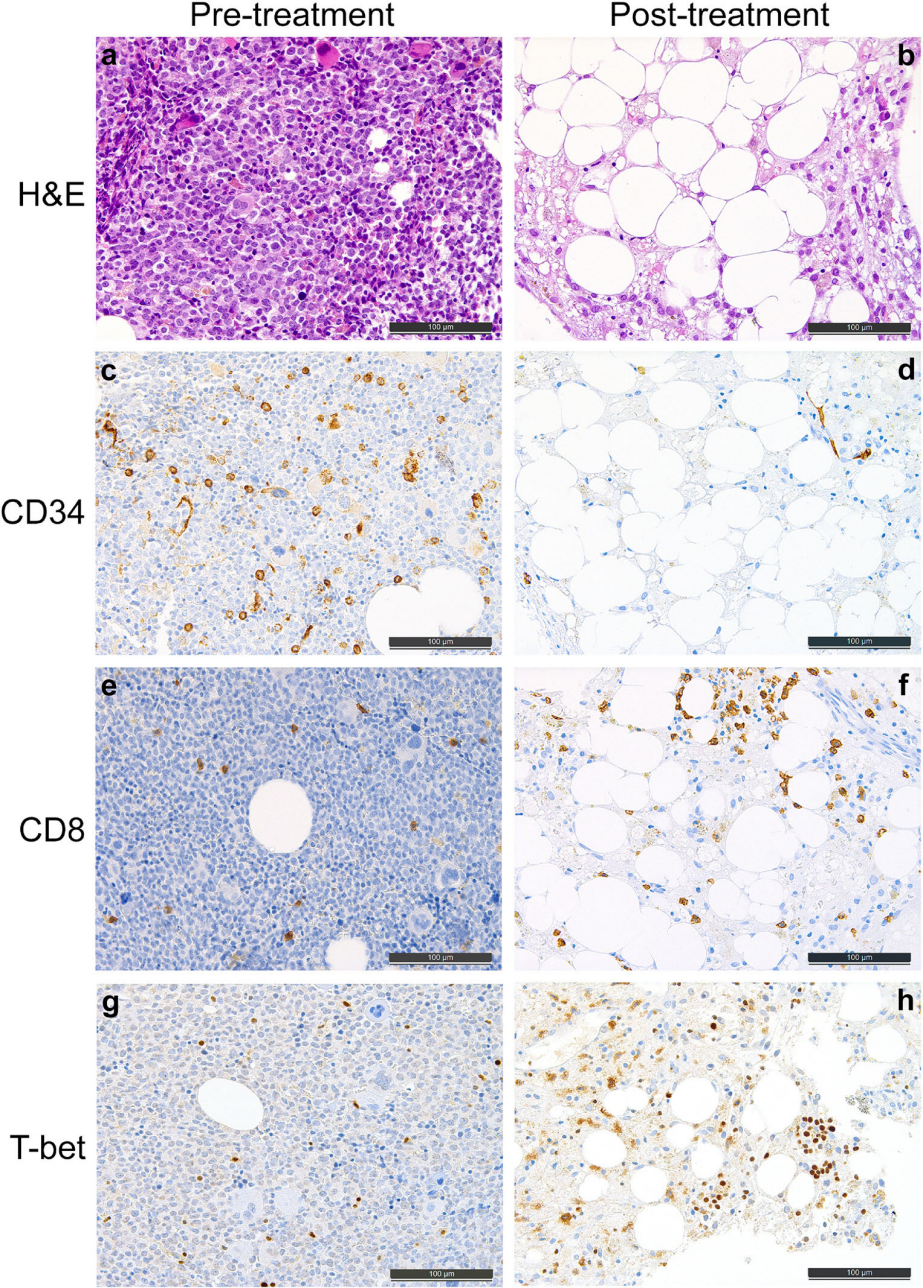
Pre- (left side) and post-treatment (right side) bone marrow biopsies of a male patient suffering from acute myeloid leukemia with myelodysplasia-related changes, who has been treated with chemotherapy and additional lenalidomide. **a** Conventional morphology at initial diagnosis, H&E. **b** Conventional morphology before the second induction cycle, H&E. **c** CD34 staining revealing higher microvessel density and partial positivity of the leukemic blasts and the dysplastic megakaryocytes before treatment, immunoperoxidase. **d** CD34 staining of the post-treatment biopsy before the second induction cycle illustrating a significant decrease of the microvessels and complete absence of positively staining blasts and megakaryocytes, immunoperoxidase. **e**, **f** Increasing amount of CD8-positive T cells from 2% pre-treatment (**e**) to 15% post-treatment (**f**), immunoperoxidase. **g**, **h** Increasing amount of T-bet-positive T-helper cells from 1% pre-treatment (**g**) to 9% post-treatment (**h**), immunoperoxidase

Irrespectively of the therapeutic regimes, the amount of blasts significantly decreased after treatment: from 45.4% (± 26.7) to 9.2% (± 21.7) in the standard arm vs. 51.9% (± 25.0) to 17.0% (± 20.8) in the lenalidomide arm (*p* = 0.001 and 0.004, respectively), (Supplementary Figure 1A-D; Fig. 2a–d); without significant difference of the drops between both treatment arms. With the decrease of blasts, morphological regeneration of the bone marrow with relative increase of adipocytes was detectible (Supplementary Figure 1A-B; Fig. 2a, b). When comparing both treatment regimens with respect to the WHO category, addition of lenalidomide was associated with a more substantial decrease of blasts, particularly in patients with AML with defining mutations (66.7% ± 14.5) compared to standard treatment (24% ± 20.2; *p* = 0.034), while in all other subgroups it was comparable between both treatment arms.

### Microvessel density

Previously, we could show that the bone marrow microvessel density (MVD; given per mm^2^), is significantly higher in newly diagnosed AML compared to healthy control individuals [22]. The current study furthermore showed a significant difference between the MVD of the various AML WHO categories (*p* = 0.011), being highest in AML-MRC (Fig. 2c) (mean 28 ± 6.8; versus 14.9 ± 10.6 in AML, NOS (Supplementary Figure 1C), versus 24 ± 10.7 in AML with defining mutations, versus 9.5 ± 2.1 in AML with defining translocations). The same was true for the drop of MVD after treatment (*p* = 0.001), showing the most prominent decrease in the WHO category AML-MRC (Fig. 2d) (mean drop 19.5 ± 17.7; versus 9.0 ± 11.6 in AML with defining mutations, versus increases in AML, NOS with 2.5 ± 9.3 (Supplementary Figure 1D) and in one evaluable AML with defining translocations with 41.0). Due to an asymmetric distribution of AML-MRC cases among the two treatment arms (i.e., most cases being treated with additional lenalidomide), the initial MVD was higher in the lenalidomide treated group (24.9 ± 11.4 versus 14.5 ± 8.5; *p* = 0.011) and – accordingly— under standard treatment microvessels increased from 14.5 ± 8.5 to 17.2 ± 12, in contrast to a reduction from 24.9 ± 11.4 to 12.8 ± 6.7 under additional lenalidomide (*p* = 0.041) (Fig. 1).

### Stem cell niches

The amount of nestin-positive stem cell niches was not modified under treatment, but their increased presence in the initial biopsy seemed to possibly correlate with an adverse prognosis regarding OS (hazard ratio: 1.75, 95% CI: 0.94–3.27; *p* = 0.076).

### Erythropoiesis

Erythropoiesis (assessed by E-cadherin) increased in both treatment arms, which was slightly more prominent with the addition of lenalidomide (4.1 % versus 9.1 %; *p* = 0.12).

### B-, T-NK- cells and monocytes

The distribution of B-, T-NK- cells, and monocytes did not differ between the pre-treatment biopsies of both therapy groups (Fig. 1). Referring to the individual WHO categories, only the presence of granzyme-B-positive lymphocytes differed among the miscellaneous groups (*p* = 0.00962), being highest in AML-MRC (mean positive cells 4.5% ± 3.9; versus 1.3% ± 1.5 in AML, NOS, versus 0.5% ± 0.7 in AML with defining mutations, versus 2.5% ± 2.1 in AML with defining translocations). Neither standard treatment nor addition of lenalidomide caused a significant increase in T cells as assessed with the pan-T cell marker CD3, but subtle changes in the composition of the T cell subpopulations could be identified (Fig. 1). Under lenalidomide, the CD4 positive T cell count remained stable, while it increased under standard treatment by 6% ± 9.1 (versus 1.9% ± 3.2 under addition of lenalidomide, *p* = 0.029; Fig. 1). The amount of CD8-positive T cells did not significantly change under therapy (Fig. 1, Fig. 2e–f, Supplementary Figure 1E-F). Importantly with respect to T cell subpopulations, T-bet-positive T-helper 1 cells seemed to slightly increase under addition of lenalidomide compared to the standard treatment (*p* = 0.063; Fig. 1, Fig. 2g–h, Supplementary Figure 1G-H). In contrast, amounts of FoxP3-positive regulatory T cells were not influenced by either treatment arm. The same was observed for CD57-positive T-large granular lymphocyte-equivalents. Remarkably, the proportion of granzyme-B-positive cells—either representing non activated (since TIA1-negative) cytotoxic T-cells or NK-cells—significantly increased under the standard regimen (6.5% ± 6.9 vs. 0.7% ± 4.6 under addition of lenalidomide, *p* = 0.019; Fig. 1). The increase of PD-1-positive T-cells was not significant in neither treatment arm, but it was slightly less pronounced under lenalidomide (0.2% ± 0.9) compared to the standard treatment (0.9% ± 1.0; *p* = 0.19).

### Cereblon

**Fig. 3.**
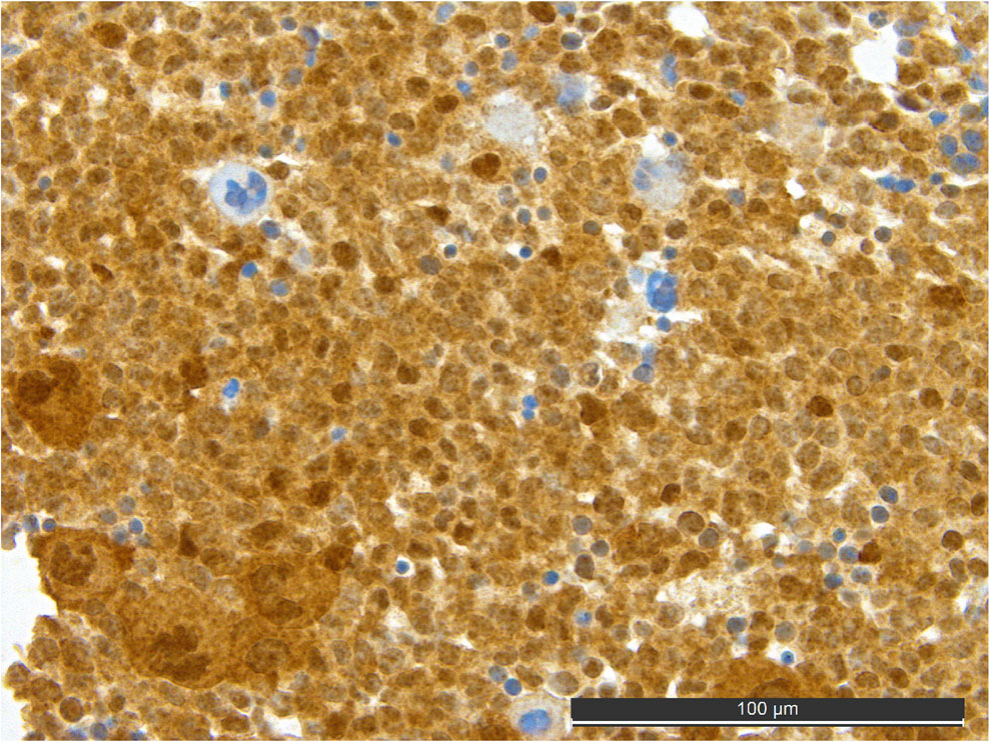
Cereblon staining of the same patient as in Fig. 2 from the time-point of initial diagnosis showing a distinct strong expression of lenalidomide’s target cereblon in all leukemic blasts and dysplastic megakaryocytes. Note: cereblon-negative residual erythropoiesis and isolated unremarkable megakaryocytes

Strong expression of cereblon in the leukemic blasts (Fig. 3) was not linked to unfavorable OS (hazard ratio: 1.18, 95% CI: 0.85–1.65; *p* = 0.328).

### Correlation analysis

The presence of T-bet-positive cells correlated with the presence of CD8-positive cells (*p* = 0.00004; *ρ* = 0.61, 95% CI 0.32–0.79), CD4-positive cells (*p* = 0.00007; *ρ* = 0.59, 95% CI 0.37-0.74), CD57-positive cells (*p* = 0.001; *ρ* = 0.51, 95% CI 0.21–0.71), and the expression of VEGFR2 (*p* = 0.002; *ρ* = 0.48, 95% CI 0.14-0.69). In turn, the expression of VEGFR2 correlated with the grade of myelofibrosis (p = 0.001; ρ = 0.53, 95% CI 0.25-0.71) and the presence of nestin-positive stem cell niches (p = 0.001; ρ = 0.53, 95% CI 0.22-0.74). Additionally, CD4-positive cells correlated with the presence of granzyme-B-positive cells (*p* = 0.001; *ρ* = 0.55, 95% CI 0.25–0.74).

### Genotype analysis of CRBN

Whether *CRBN* was homozygous wild type (26 instances) or contained variant alleles (8 instances), did not have an impact on the treatment effect or the prognosis, irrespective of the therapy applied.

### Outcome

The median OS of the cohort was 18.5 months (95% CI 8.–-46.8, 30 events) and the median PFS 8.9 months (95% CI 6.4–17.1, 34 events). Neither the OS, nor the PFS differed significantly between the two treatment arms, in accordance with the results of Ossenkoppele et al. (12). Neither age nor gender were relevant for clinical outcome. PFS was significantly influenced by the karyotype (*p* = 0.012) and highly significantly by the WHO category (*p* = 0.0061), being shortest in AML-MRC. In this category, the median PFS differed between the patient, who received standard treatment (0.4 months), and the remaining patients receiving additional lenalidomide (2.6 months; *p* = 0.25), yet this did not reach statistical significance. As expected, the karyotype (*p* = 0.0042) and the WHO category (*p* = 0.0030) had a significant impact on the OS. Neither the amounts nor the dynamics of the various studied T cell subpopulations correlated with prognosis.

## Discussion

Despite extensive research efforts, the prognosis of AML—especially in the elderly—remains poor. Therefore, more effective and better tolerable therapeutic strategies are of urgent need. Lenalidomide, an immune-modulatory drug already successfully implemented in other hematological malignancies associated with intrinsic dysfunction of the bone marrow, gathered attention as a potentially effective drug in AML. Indeed, in an AML murine model, immune-modulatory drugs were shown to hamper leukemia progression in vivo and to induce enhanced allogenic NK-cell activity [23].

In this translational research study of the Swiss cohort of HOVON103 AML/SAKK30/10, we were able to show perceptible differences in the outcome of AML-MRC patients: those with additional lenalidomide treatment had a longer PFS compared to the AML-MRC patients treated with chemotherapy alone. Unfortunately, a statistical significance was not reached due to the low case numbers and the unfavorable random distribution of patients between both treatment arms (only one patient received standard chemotherapy and 7 patients received additional lenalidomide). Nevertheless, patients with AML-MRC had a significantly higher bone marrow MVD compared to other AML categories and the most prominent lenalidomide-induced decrease of microvessels. Since it is known that a high bone marrow MVD is associated with a poor prognosis and that the reduction of microvessels correlates with treatment response [24, 25], we hypothesize that this particular patient subgroup, i.e., AML-MRC, might profit from the antiangiogenic effects of lenalidomide. Consistently, AML blasts are known to depend on the presence of nestin-positive stem cell niches [26], the density of which in turn correlated with the expression of the neovascularization-promoting VEGFR2 in our cohort. In general, myeloid malignancies such as AML can remodel stem cell niches to support malignant growth and to sustain stemness [27, 28]. Concordantly, in our study, therapy-induced blast reduction was not reflected by numeric changes of nestin-positive stem cell niches, yet an increased presence of such niches was linked to adverse outcome with respect to OS. If these niches represent a treatment-refractory place of retreat of leukemic blasts remains to be determined.

At least in our study, the effect of lenalidomide in AML-MRC was independent of the presence of del(5q), which has been linked to a better susceptibility to lenalidomide treatment in MDS [29]: the only patient of our cohort, who displayed del(5q) (in the context of a complex karyotype) and fulfilled the criteria of AML-MRC, received standard therapy. Irrespectively of the AML category, subtle treatment-induced changes in the composition of T cell subpopulations were observed, although the total number of T cells did not significantly differ between pre- and post-treatment biopsies. Under lenalidomide, the amount of T-bet-positive T cells more consistently increased, which might be interpreted as a sign of increased T cell driven immune response against the tumor cells. Indeed, the transcription factor T-bet has been found to be one of the key players in the induction of leukemia-reactive T-cells, and lower T-bet expression rates have been linked to poor immune responses and disease progressions [30, 31]. Correspondingly, the presence of T-bet-positive cells correlated, among others, with the presence of CD8-positive cytotoxic T cell-equivalents and CD57-positive large granular lymphocyte-equivalents.

The low PD-1-positive T cell count in all our samples fits to the fact that immune-checkpoint inhibitor treatment failed to achieve a major breakthrough in AML. This may be linked to a lower immunogenic potential compared to solid tumors such as melanoma or non-small cell lung cancer or due to genuine impairment of the antigen processing machinery in AML [32]. Nonetheless, we found a slight increase of PD-1-positive T cells in the bone marrow biopsies after treatment, indicative for a growing T cell exhaustion and enhanced inhibition of anti-tumor immune response. Although not significant, this effect seemed to be less pronounced under lenalidomide, which leads us to hypothesize, that lenalidomide—as an IMiD—may support some anti-leukemic immune responses. This is in accordance with observations in other hematologic malignancies such as multiple myeloma, in which lenalidomide significantly reduces PD-1 surface expression on T cells and enhances the anti-tumor response [33]. Additionally, lenalidomide was noticed to counteract the negative impact of PD-1-positive cells in follicular lymphoma patients, potentially due to its stimulating effect on the immune response [4].

The presence of cytotoxic, granzyme-B-positive T cells differed between various AML categories of our study collective, being highest in AML-MRC. This observation is supported by recently published data, demonstrating an association between cell-intrinsic genetic alterations in AML and the amount of cytotoxic lymphocytes, suggesting that AML-MRC may be more immunogenic. Indeed, genetic alterations linked with poor prognosis [*TP53*, del(5q), complex karyotypes] and being more frequently encountered in AML-MRC, as well as AML-MRC per se were found to be associated with higher T cell induced cytolytic activity [34].

Significant decrease of leukemic blasts under treatment was observed in both arms. With respect to the AML-categories, a more substantial blast drop under lenalidomide was noticed in AML with defining mutations, despite the fact that the presence of driver mutations such as *FLT3* and *NPM1* has been linked to low cytolytic activity of the tumor microenvironment [34]. If this effect is linked to the immune modulatory or other functions of lenalidomide and if it is reproducible in other AML collectives, remains to be determined [35].

Strong expression of lenalidomide’s target cereblon in the leukemic blasts was rather associated with an unfavorable OS, which has also been documented for gastric marginal zone lymphomas [36], but has until now not been addressed in myeloid neoplasms and may deserve attention in larger studies. Interestingly, all of the described effects were independent from the genotype of the cereblon gene (*CRBN*), which is in line with data from other IMiDs [23].

Our study has several shortcomings. We were not able to investigate the bone marrow samples of all patients included in the HOVON103 AML/SAKK 30/10 study due to lacking material and therefore the sample size is very limited. Due to the random distribution of cases, there was an imbalance of the assigned treatment arms among the AML categories. Finally, the observed beneficial effect of the addition of lenalidomide was present only in a subgroup of patients, i.e., AML-MRC, which although being a WHO category, is yet a post-hoc subcohort from the perspective of the initial clinical trial design [12].

Observable on a small number of patients, addition of lenalidomide led to a perceptible but not significant increase of PFS in patients with AML-MRC, a category characterized by a poor prognosis and often complex karyotypes. Our findings are in keeping with encouraging results in the literature, showing on the one hand a direct anti-leukemic effect of lenalidomide, and, on the other hand, an important immune-activating impact on the tumor microenvironment. We think that our observations also highlight the importance of taking the WHO defined subentities into consideration when designing clinical trials in AML.

## Supplementary Information

Supplementary Figure 1 Pre- (left side) and post-treatment (right side) bone marrow biopsies of a female patient with acute myeloid leukemia, not otherwise specified who has been treated with chemotherapy only. **A:** Conventional morphology at initial diagnosis, H&E. **B:** Conventional morphology before the second induction cycle, H&E. **C:** CD34 staining revealing initial microvessel density and distinct positivity of the leukemic blasts before treatment, immunoperoxidase. **D:** CD34 staining of the post-treatment biopsy before the second induction cycle illustrating a profound decrease of CD34-positive blasts and constant microvessel density, immunoperoxidase. **E and F:** Pre-treatment amount of CD8-positive T-cells (8%) (E), which slightly decreased after chemotherapy to 5% (F), immunoperoxidase. **G and H:** Increasing amount of T-bet-positive T-helper cells from 1% pre-treatment (G) to 4% post-treatment (H), immunoperoxidase. (JPG 6819 kb)

ESM 2 (XLSX 18 kb)

## Data Availability

The datasets generated during and/or analyzed during the current study are available from the corresponding author on reasonable request.
