# Supplementary figures and images for: Effects of lenalidomide on the bone marrow microenvironment in acute myeloid leukemia: Translational analysis of the HOVON103 AML/SAKK30/10 Swiss trial cohort
Source: Ann Hematol. 2021 Mar 2;100(5):1169–79. doi: 10.1007/s00277-021-04467-2 (PMC8043896; doi:10.1007/s00277-021-04467-2)

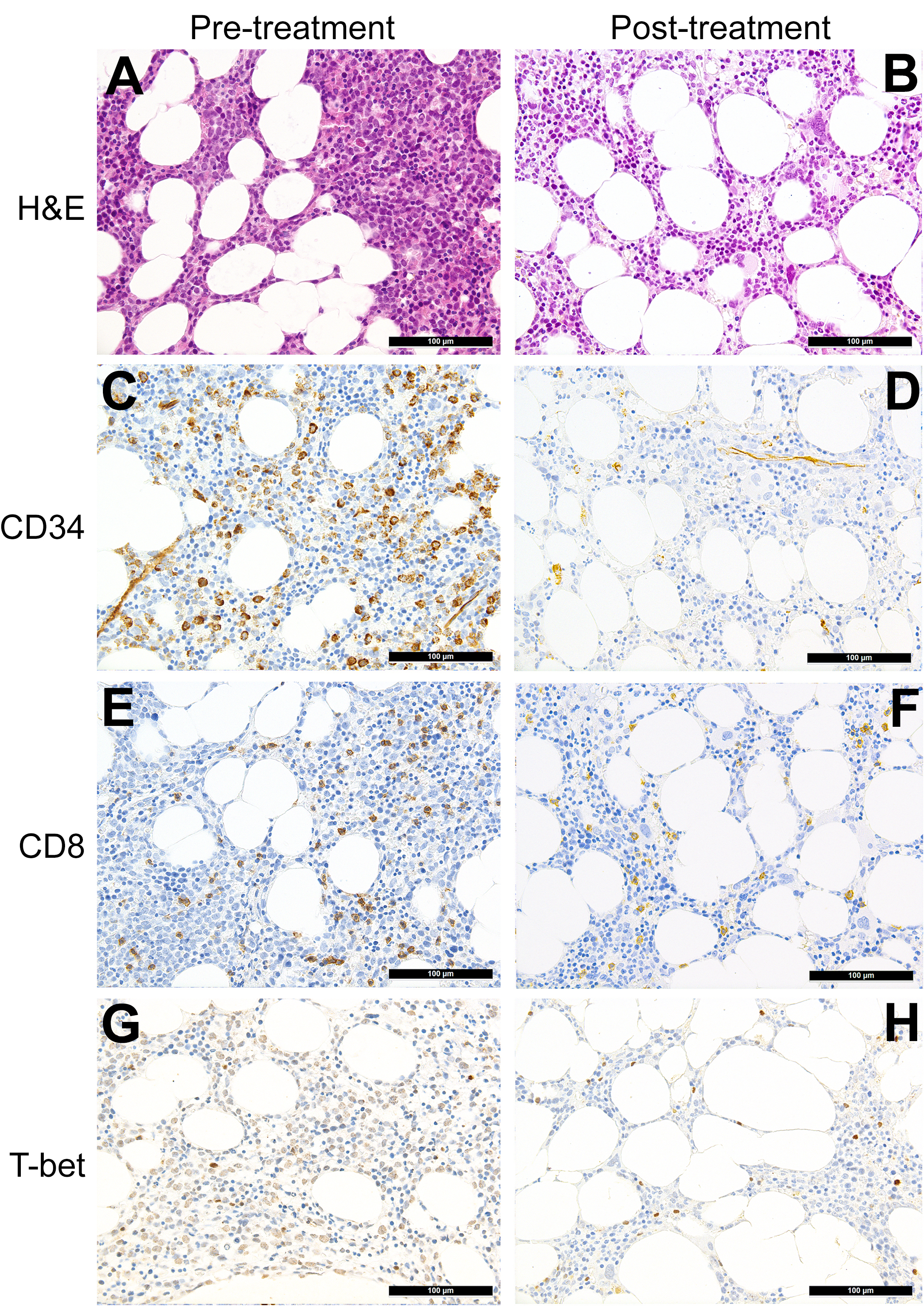

Supplement: Supplementary file 1 — Pre- (left side) and post-treatment (right side) bone marrow biopsies of a female patient with acute myeloid leukemia, not otherwise specified who has been treated with chemotherapy only. A: Conventional morphology at initial diagnosis, H&E. B: Conventional morphology before the second induction cycle, H&E. C: CD34 staining revealing initial microvessel density and distinct positivity of the leukemic blasts before treatment, immunoperoxidase. D: CD34 staining of the post-treatment biopsy before the second induction cycle illustrating a profound decrease of CD34-positive blasts and constant microvessel density, immunoperoxidase. E and F: Pre-treatment amount of CD8-positive T-cells (8%) (E), which slightly decreased after chemotherapy to 5% (F), immunoperoxidase. G and H: Increasing amount of T-bet-positive T-helper cells from 1% pre-treatment (G) to 4% post-treatment (H), immunoperoxidase. (JPG 6819 kb) [file 277_2021_4467_MOESM1_ESM.png]
